# Mr. Guthrie on the Diseases and Injuries of Arteries, &c.

**Published:** 1830-07-01

**Authors:** 


**ON THE DISEASES AND INJURIES OF ARTERIES, WITH THE OPERATIONS REQUIRED FOR THEIR CURE; BEING THE SUBSTANCE OF THE LECTURES DELIVERED IN THE THEATRE OF THE ROYAL COLLEGE OF SURGEONS, IN THE SPRING OF 1829. By *G. J. Guthrie*, F.R.S. Professor of Anatomy and Surgery to the Royal College of Surgeons, &c. &c. 8vo. pp. 408. Burgess and Hill. London, 1830.**

ON the importance of the subject of aneurism and wounds of the great vessels of the human body, we need scarcely dwell. It is to surgery what fever is to physic, nay more, it is almost its alpha and omega. On an intimate acquaintance with the physiology and pathology of the arteries depends the doctrine of inflammation, that essential dogma which has so long and so often convulsed the schools, which has raised to power and shaken to their foundation the successive dynasties of Humorist and Solidist, the tyrannies, if we may use such an expression, of the great masters, of Boerhaave, of Cullen, and of Hunter. The surgeon who is acquainted with the nature of the diseases and accidents to which the arteries are liable will certainly prove a benefactor to humanity; the man who is not so and yet has the responsibility of such cases imposed on him in practice, is as certainly a curse. Who can peruse the serio-comic anecdotes in the works of John Bell, without feeling a shudder creep over his mirth at the satiric picture of an ignorant surgeon, so happily delineated by the powerful graver of that able man! Fortunately the objects of such satire are daily becoming more and more scarce, and the great body of the profession are possessed of a general degree of information on these topics which pleasingly contrasts with the ignorance of "sixty years since."

Mr. Guthrie's work is divided into two parts, not altogether unconnected, but yet in many points distinct. The first comprises the diseases of arteries, the second their injuries. The object of the former is, according to our author, "to demonstrate the value and importance of that portion of the pathological collection in the museum of the Royal College of Surgeons which relates to the subject of aneurism; and to prove that the labours and researches of Mr. Hunter anticipated nearly all the observations which have been made by his contemporaries and successors." In the latter Mr. G. has entered more fully into the consideration of the nature and treatment of wounds of arteries, and illustrated these points by observations and cases which occurred during the late war in Portugal, Spain, France, and the Netherlands. The matter contained in the volume has been for many years announced as preparing for the press, but although it has not hitherto appeared in print, it has been annually promulgated in our author's surgical lectures; and if delayed it has certainly not been withheld. Our narrow limits not permitting us to notice the whole, nor even the greater part, of the work in the present number, we shall pass at once to the subject of wounds and injuries of arteries, and reserve that of aneurism for another and early opportunity.

Mr. Guthrie commences with a remark of much value, though too often neglected by physiologists and surgeons, viz. that the analogy between wounds of the arteries in animals and in man, however specious, is not satisfactory; because neither are the consequences similar, nor is the reparative process adopted by nature the same. We all know with how much difficulty aneurism is produced in the lower animals, with how much facility it occurs in the human species. Nature is all powerful in arresting hæmorrhage in the former, but no judicious surgeon will trust to her efforts alone in the wounds of arteries in the latter; here art must come to the assistance of Nature in the most effective manner that the state of the case will permit. After noticing briefly but succinctly the phenomena observed to succeed the infliction of wounds on the arteries in man and other animals, and giving an abregé of the opinions of writers, on the steps pursued by Nature for the suppression of hæmorrhage, from Galen and Celsus down to Dr. Jones, Mr. Guthrie proceeds to deliver his own opinions.

"I must observe, in regard to the observations which follow, that they have been deduced from others made on man, suffering from different states of injury, which the opportunities offered to me during the Peninsular war allowed me to make on an extensive scale. Some points have been corroborated by experiments made on animals; and I shall here acknowledge my obligations to Mr. Sewell, of the Royal Veterinary College, and to Mr. F. Thomson, who undertook several of them, and gave themselves much trouble to ascertain the objects I had in view.

In the different theories I have noticed, and especially in that of Dr. Jones, it does not appear that the gentlemen who proposed or maintained them have ever conceived that there was a difference in the means employed by nature, according to the size of the artery injured or divided; that the difference of structure between an artery, such as the carotid or the inguinal, and the tibial or the radial, could cause any deviation from the process they described as taking place, and as they presumed in one invariable manner in all arteries. I shall venture however to say, that on the size and variation of structure of the artery, the process employed by nature essentially depends; that it is not the same in large, as in small arteries; and that it is not even quite the same in the upper and lower ends of the same artery.

An artery of moderate dimensions, such as the tibial or brachial, and particularly all below these in size, are in general capable by their own intrinsic powers of arresting the passage of the blood through them without any assistance from art, or from the surrounding parts in which they are situated. This overthrows at once the whole theory which relates to the sheath of the vessel and its offices, and in a great measure to the importance derived from the formation of an external coagulum." 223.

As it mostly happens that patients do not choose to bleed to death for the pure love of science, nor surgeons avoid restraining hæmorrhage on their own or their patients' accounts, so we seldom have an opportunity of seeing the processes of nature in human beings, undisturbed by the interference of art. Mr. Guthrie, however, deems it odd ratiocination to bleed an animal until it dies, in order to determine how a bleeding was suppressed, which in point of fact was never suppressed at all.

"In my work on Gunshot Wounds, I have related the case of a soldier, who had his arm carried away by the bursting of a shell at the seige of Ciudad Rodrigo, and who was brought to me shortly afterwards. The axillary artery becoming brachial was torn across, and hung down lower than the other divided parts, and pulsated up to the very extremity. Pressed and squeezed in every way between my fingers in order to make it bleed, it still resisted every attempt, although apparently by the narrowest possible barrier, which appeared to be at the end of the artery, and formed by its contraction. The canal was marked by a small red point, to which a very slight and thin layer of coagulum adhered, the removal of which had no influence on the resistance offered by the end of the artery to the passage of blood through it. In another case of a similar character, I cut off the end of the artery at less than an eighth of an inch from the extremity, when it bled with its usual vigour. In both, the vessel for that distance was contracted, so as to leave little or no canal at its orifice, and what there was, was filled by a pin-shaped coagulum." 224.

If in amputation at the wrist the radial or ulnar arteries be allowed to bleed until they cease to do so, the jet subsides into a stream, and the latter gradually diminishes until it ceases altogether; the extremity of the vessel being covered by a layer of coagulum of greater or less thickness. Some retraction of the artery must take place in such cases, but it cannot be fairly estimated. On examination after death or amputation the contraction of the vessel is evident, as well as the formation of a very slight external coagulum, extending into the canal of the artery. In these cases the sheath of the vessel can do nothing, for there is none; nor does any internal coagulum, strictly speaking, exist; nor in the instance of such moderate sized arteries does the diminished power of the circulation go for much. Although he has thus established the fact, that second-rate arteries in the extremities will cease to bleed, without the assistance of surrounding parts, Mr. Guthrie by no means intends to assert that they cannot and never do receive any; on the contrary, he is aware that in a great number of instances the reverse is the fact.

After remarking that the power of the heart over the circulation is egregiously over-rated, Mr. G. asserts without fear of contradiction, that when a large artery is fairly exposed and divided, a very slight degree of pressure perpendicular to its orifice is sufficient to suppress the hæmorrhage; and an equally moderate pressure on the sides of the artery will prevent the passage of any blood through it. If, however, the vessel be only half cut through, and in a situation inaccessible to slight but equable pressure, the hæmorrhage continues in spite of all attempts at its suppression. When the femoral artery is cut across in the upper part of the thigh, whether it be done by a cannon shot, musket ball, or knife, the patient does not always bleed to death, although frequently lost in consequence of the injury. If the division of the vessel take place in the middle or lower half of the thigh, the bleeding will probably cease of itself, and if it recurs it is more likely to proceed from the lower than the upper portion. Such are the statements of Mr. Guthrie, and no doubt they are founded on actual observation in the field. We doubt, however, whether surgeons in civil practice are generally of the same opinion, perhaps, as Mr. Guthrie has elsewhere remarked, because a little experience and more alarm, have conspired to persuade them, like the fat and fearful knight, of the reality of the terrors of their own conjuration. However this may be, it is evident that Mr. Guthrie looks upon these injuries without any degree of dread or consternation. At the battle of Toulouse, a large shot struck an officer in the thigh, and divided the artery about three inches below Poupart's ligament. Circumstances prevented immediate attention, and he died without any operation being performed. Desirous of seeing by what means the hæmorrhage "had been suppressed," our author carefully removed the artery, and found that its divided end was irregularly torn, and filled up by an external coagulum, which was slightly compressed at its inner end by a trifling contraction of the artery that served to keep it in its place. The rest of the coagulum filled the hollow in the sheath which the retraction of the artery had occasioned. "In this case," says Mr. G. "so unlike those I have hitherto noticed, the first natural cause giving rise to the suppression of the bleeding was the diminution of the power of the circulation; the second, the formation of a coagulum, formed in the hollow of the sheath left by the retraction of the artery. Contraction had done nothing." In a similar case, at the battle of Salamanca, the hæmorrhage stopped and did not return, but the patient died exhausted; no operation whatever was attempted, nor tourniquet applied. The artery, on examination, was in just the same state as the former, with the mere exception of the orifice being a little more contracted, and the external coagulum less in size, and projecting like a mamillary process. In other instances of a like description the appearances have more or less resembled the above; unless where the patients had died immediately, when the torn extremities of the artery have been quite open, with very little or no surrounding coagulum. It must be obvious to our readers that the persons whose cases have been related were similarly circumstanced to those of animals bled to death, and consequently the appearances resembled those observed by Dr. Jones in his experiments.

Mr. Guthrie has had many cases of injury of the femoral artery from smaller projectiles under his care. When the artery has been completely cut across in the *middle or lower part* of the thigh, the patient has either died without assistance, or the hæmorrhage has ceased spontaneously. He has not met with an instance in which it has been necessary to tie the femoral artery after it had been divided and the hæmorrhage had ceased for the space of twelve hours, the efforts of nature being efficient to prevent its return. Mr. Guthrie has met with a considerable number of cases of gangrene of the extremity, or hæmorrhage from the lower end of the vessel requiring amputation, after wound of the femoral artery. Ten or eleven cases of this kind are detailed by Mr. G., but as many, if not most of them, have already been published in the 4th vol. of the Medical and Physical Journal, and as on several occasions we have drawn the attention of our readers to this point, we shall pass them over without any further comment.

"An artery of the size of the femoral at the *middle* or *lower* part of the thigh, retracts on being divided within its sheath ; this retraction is also accompanied by a contraction of the orifice or extremity, which gradually assumes the shape of a Florence oil flask, or French claret bottle, in a similar manner to the contraction of the axillary artery, described page 224. I have not met with an instance so perfectly clear and decided of the femoral artery hanging out of a wound as in this case of the axillary artery, so as to demonstrate that the whole process is carried on in a similar manner. I have however seen the femoral artery at the lower part of a thigh, which had been struck by a cannon ball, so little supported by coagulum, and yet so much closed, as to lead to the belief, that in some instances the extremity of it may be closed by similar means, a conclusion which analogy would lead us to, if observation were wanting. In all successful cases, the retraction of the artery leaves a space occupied by a coagulum, which also in an artery of this size fills up the contracting opening, which is in a circular direction, just within the ragged edges, which when they exist do not themselves contract, because the continuity of fibre is wanting. The continued contraction of the artery expels the external coagulum, and this operation is assisted by the lymph effused from the cut edges and from the coats of the vessel; so that in a few days the whole of the coagulum is removed with the purulent discharge from the part ; and the place it would occupy, the orifice of the artery, and the surrounding parts for at least an inch in extent, are filled up and covered by a yellowish green-coloured matter, very distinct in appearance from the neighbouring parts. On the examination of a wound after death or amputation, in which it was known that a great artery had been divided, I have always from this appearance pointed out the situation of the extremity of the artery.

The contraction of the divided end of the artery is confined in the first instance to its very extremity, so that the barrier opposing the flow of blood is formed by this part alone, as I proved by cutting it off in the case mentioned, page 224. This contraction goes on however increasing for the space of an inch, and the inside of this contracted inch of the vessel is filled up with an internal coagulum, which takes the shape of and adheres to the inside of the artery, rarely extending as far as a collateral branch, or under almost any circumstances beyond a couple of inches. Towards the extremity of the artery it adheres firmly, so as to form a real substantial obstacle to the flow of blood through it. The very orifice of the artery on the outside of this is covered by the yellowish green-coloured matter or lymph, which ultimately becomes organized. These processes are continued long after the wound is healed. The artery generally goes on diminishing and contracting up to its first large branch, so that of four or five inches, two or three will be impervious, the remaining part very much contracted, although perhaps still permeable by a probe. The accompanying nerve, where there is one, has just done the reverse, the cut extremity having become enlarged or bulbous, and gradually diminishing as it is traced upwards, until it becomes of its proper size." 248.

Mr. Guthrie observes, that it is a very curious and interesting fact, that the lower end of a divided artery is more prone to secondary hæmorrhage than the upper; so much so indeed, that when it occurs after having been arrested for a period of four hours, it takes place in all probability from the lower end. This may always be known by the darker colour of the blood, and by its *welling* out in a continuous stream without any arterial impulse. Mr. G. paid particular attention to this point during the war, and he is confident he cannot be mistaken as to the fact.

"The same kind of yellowish green matter marks and covers the situation of the lower extremity of the artery, as it does the upper; it is, however, thinner where it immediately covers the end of the artery, which in none of these cases was contracted in the conical manner I have described as occurring in the upper extremity of the vessel. On the introduction of a probe into the artery with the greatest gentleness from below, it made its appearance at a point on the yellow space, raising a thin portion of it as it protruded. On laying open the artery, the orifice seemed to have been once closed by this layer of fibrine or lymph, but without a degree of contraction corresponding to that observable in the upper end of the same artery; the layer still forming an obstacle, sufficient to cover and close three-fourths of the orifice, the blood having flowed through the remaining fourth." 250.

These appearances seem to indicate a different process to that adopted for the closure of the upper end of the vessel, and their frequency to demonstrate that the process is a natural one. Two cases are detailed in illustration of the foregoing statements, and we shall briefly glance at the first.

Serjeant Lillie, æt. 32, was wounded in the thigh by a musket-ball, which described a track of seven inches, and was extracted behind on the field. He bled a good deal at the time, but restrained the hæmorrhage with his sash, and, for 19 days, the wound appeared to be going on extremely well, when, on making a sudden turn in bed, dark-coloured blood flowed from both orifices of the wound in considerable quantity. Mr. Dease, in the absence of Mr. Guthrie, performed the operation for aneurism at the lower part of the upper third of the thigh; in 8 days the hæmorrhage returned, the limb was amputated, and the patient died. On examination, the artery was found to have been divided, where it passes between the tendinous expansion of the triceps and the bone; the upper portion, divided by the shot, was closed; a probe introduced into it from above would not come out at the face of the wound, although the impulse given to the part on moving it was observable in the middle of a large yellowish green spot, where the vessel presented a claret-shaped contraction for about an inch, and an internal but small coagulum for nearly the same extent. The lower or bleeding end of the artery was marked by a nearly similar spot; but, on passing a probe upwards from the popliteal space, it came out at a very small hole in the extremity of the artery in the centre of the spot. The canal of the vessel was not contracted or diminished, but only apparently closed by a layer of the yellowish green lymph laid over it, and adhering to its circumference.

When an artery is merely cut or torn, without being completely divided, it is just as if it had given way by ulceration. It can neither retract nor contract, but, if pressure be not accurately applied and maintained, it will bleed until the patient is destroyed. If the vessel is a small one, as the temporal artery, it must be cut across; if of larger dimensions, a ligature should be placed on it above and below the wound, between which it may or may not be divided, at the pleasure of the surgeon. This rule is so important that every tyro should learn it by heart. We now arrive at the section entitled—

### ON THE METHODS OF PERFORMING OPERATIONS ON WOUNDED ARTERIES.

Our author sets out with the just and important principle, that however applicable may be the Hunterian operation to cases of aneurism, and however brilliant its success in the treatment of that disease, it is totally inapplicable to wounded arteries. Surgeons for some time imagined that the same operation must answer in the one which had been followed by such splendid consequences in the other, and dazzled by the glory that surrounded the genius of Hunter, they misconstrued his views and perverted his principles. The error is now perceived and abandoned, but although the necessity of securing an artery of any size above and below the wound in its coats is now generally acknowledged, Mr. Guthrie contends that the modus operandi has been absurdly and unnecessarily retained. The examples of this mistake singled out by Mr. Guthrie are, the operation for wound of the posterior tibial artery, of the axillary, and ulnar at its origin from the brachial. In operations for aneurism, the surgeon, in some measure, chooses his situation, and proceeds in a straight-forward manner, according to certain definite rules. In casual wounds of course it must be otherwise, but Mr. Guthrie contends that rules are still inflicted to clog and bewilder the younger surgeon. "The principal error," says our author, "in this method of proceeding, as adapted to wounded arteries, arises from a strange and unaccountable fear of cutting muscular fibres, which seems to have pervaded the minds of all the surgeons of the present day who have treated on these subjects."

Suppose, for instance, that the posterior tibial artery is wounded, and the surgeon determines to tie the vessel, he will be obliged, according to the usual mode of operating,* to raise the inner edge of the gastrocnemius muscle, to detach the inner head of the soleus from the tibia, to divide the deep-seated fascia on a director, and then to secure the vessel in a deep cavity, taking care to avoid the posterior tibial nerve. After noticing, in a very forcible manner, the acknowledged difficulties of this awkward operation, Mr. Guthrie proceeds to propose his own method of operating:—

"An incision is to be made six or seven inches in length, by successive and rapid incisions, through the integuments and muscles of the calf of the leg down to the fascia. The centre of the incision is to be on a line with the shot holes, or if they are diagonal to each other, between them; and it may be either directly in the middle of the calf, or a little to the side of, or directly over, the artery supposed to be wounded; it is not material which. The smoothness of the fascia points it out, and the loose cellular membrane connecting the divided muscles to it, allows of the edges of the long incision being easily separated, and to such a distance as to admit of the exposure of the great nerve, the arteries, and veins, in as distinct a manner as any other arteries, veins, and nerves, can be exposed in the human body. The tourniquet is now to be unscrewed, and the bleeding, if the wound did not bleed before, leads to the spot where the artery is injured. The knife may be applied perpendicularly to the fascia, and the artery laid bare for three or four inches in extent, by as common a piece of dissection as any ever practised, and nothing can interrupt the application of the ligature. The nerve and the fascia cease to be surgical bugbears, and the operation is as simple as any in surgery. No surgeon or anatomist will dispute this statement: he may however say, that the muscles have been divided, and that surgeons have not been in the habit of cutting through them by a fair incision in their length; that they have hitherto only done it by insinuating a director under the head of the soleus, and separating it from its attachment to the bone; as if the separation of a muscle from its bony attachment was not much more likely to lead to weakness and defect in the action of that muscle, than a mere interstitial incision in a longitudinal direction. There is no anatomist who will deny that it is so." 261.

In order to prove that a muscle may be cut across, whenever it may be desirable to do so in order to place a ligature upon an artery, and that little or no inconvenience is the consequence, Mr. Guthrie relates the case of Lieutenant Colonel Wildman, in whom the deltoid was completely divided by a sabre-cut. By raising the arm to a right angle with the body, and bringing the sides of the wound together with compress and bandage, granulations sprung up, and the officer's recovery was so perfect, that he is now unconscious of any defect in strength and motion. In a French soldier, also, the lower and fore-edge of the pectoralis major was completely cut across, and yet merely a little weakness, of no consequence was left. In accordance with these facts and the principles already brought forward, Mr. Guthrie criticises sharply the operation, as laid down in Harrison, of tying the axillary below the clavicle, for wound of the artery through the pectoralis muscle. He also animadverts on a case related in Mr. C. Bell's Commentary on John Bell's Surgery, in which this operation was performed unsuccessfully for secondary hæmorrhage from the axillary, after the arm had been torn off by machinery. The artery, when

* For an account of this operation, see Harrison on the Arteries.

wounded, should always be secured at the spot, and, if necessary, the pectoralis major muscle should be divided, taking the hole or cut as the centre of the incision.

Mr. Guthrie next passes on to the manner of securing the ulnar artery when wounded a little below its origin, and whilst covered by the pronator teres, &c. In Mr. Harrison's work it is stated that, at this point, a ligature of the artery "would be impracticable," but Mr. Guthrie tauntingly remarks, that it would only be so because it is so considered. The surgeon should make a clean incision down to the artery through all the muscular fibres that cover it, avoiding the median nerve as it runs between the two origins of the pronator teres, and then he should place a ligature above and another below the wound in the artery, "when there would be nothing more to do." Mr. Guthrie has seen these parts divided, and he has divided them himself, and the patient has recovered without any sensible defect. In a case at the battle of Vimiera, in which the ulnar artery was wounded, Mr. G. cut down upon the vessel, which he found more than half divided, and tied it above and below the wound. The patient was cured. Before continuing the thread of our analysis, we may observe that Mr. Guthrie has proved, that the mere division of muscular fibres is far from being an insuperable objection to an operation. The only question appears to be, the facility in all cases of performing it. Suppose, for instance, a wound of the ulnar artery near its origin, with great extravasation of blood into the neighbouring parts; in such a case, it would be difficult to find and secure the bleeding vessel, buried, as it is, under layers of muscle and a mass of blood. The same may be said of wound of the posterior tibial artery; but, as Mr. Guthrie has had a practical experience in these operations which others have not, his opinion is entitled to more weight than theirs. We would not be understood as quarrelling with the principle inculcated, of tying both ends of a wounded vessel in every practicable case; on the contrary, it is one to which we yield our undivided assent, and to which we would most earnestly direct the attention of our readers.

Connected with this subject our author adverts to, and keenly animadverts on, a memoir published by M. Dupuytren, in the Repertoire General d'Anatomie et de Physiologie, &c. tome v. 1828, entitled—"Sur les Anévrysmes qui compliquent les Fractures et les Plaies d'Armes-à-Feu, et sur leur Traitement par la Ligature, pratiquée suivant la Methode d'Anel."

After alluding to some cases by Petit, Pelletan, M. Delpech and himself, M. Dupuytren proceeds to relate, at some length, the case of M. de Gambaud, a captain of cavalry, who received a wound from a horse-pistol bullet, which entered the upper part of the right leg, from the front backwards and from the outside inwards, passing between the tibia and the fibula, which latter it slightly injured. Violent bleeding immediately ensued, which was stopped by compress and bandage, although the limb became swollen and very painful. An aneurismal tumour formed, the pulsations of which were at first arrested by the pressure of a tourniquet and pad on the femoral artery, but they soon returned, and, on the thirteenth day, hæmorrhage took place from the wound. The bleeding returned from day to day, and M. Dupuytren was called into consultation with Messrs. Aumont and Dessien The foot and leg were of a violet colour, swollen, cold and numb, and uncertain whether the anterior or posterior tibial artery, or the peroneal or popliteal, or several of them at the same time, were divided, M. Dupuytren tied the femoral artery. Inflammation was moderate; on the twentieth day the ligature on the femoral artery came away, and, in six weeks, all the wounds were completely healed.

"'Ought we to attribute'" says M. Dupuytren, "'the success of this operation to the accidental concurrence of fortunate circumstances? or ought we look upon it as the natural and necessary consequence of the principle acted upon, in placing a ligature on the femoral artery? and should such a method of proceeding be established as a precept in surgery? To answer these questions, allow me again to mention, that this method of treating simple aneurisms always stops the pulsation of the tumour; and even when employed against aneurism complicated with fracture has been very successful; and, finally, that this method, which M. Delpech and myself first practised nearly at the same time, in cases of hemorrhage following amputation, has invariably been attended with success. From these results I think it evident, that the success of the present operation was not dependent upon any fortuitous occurrence; but on the contrary was the natural consequence of the practice pursued. The ligature, in suspending the course of the blood in a divided vessel, the solution of continuity of which had caused an external and internal bleeding, gave time and means to the inflammation to cicatrize the wound in the vessel, and to render the cut extremities impermeable to the blood which the anastomosing branches might bring to them.

To judge by analogy, this obliteration ought to be more easy and more certain after gunshot wounds than any other.

One of their most remarkable effects being to contract *(froncer)* the orifices of the vessel, to concrete or coagulate the blood contained in their extremities, and to render them impervious.

Without therefore wishing to elevate this single fact into a principle, I do not hesitate to consider the success obtained in this case of M. de Gambaud as the forerunner of other similar fortunate results.

Many other reflections occur to me, but I hasten to a conclusion, drawing attention to the two principal points of the memoir. First, the rupture of the principal artery of a limb, occasioned by a fracture, and followed by an extravasation of arterial blood round the broken bone. Secondly, the rupture of the principal artery of a limb caused by a musket ball, followed by an extravasation of arterial blood, having in both cases the character of an aneurismal tumour. This complication of injuries, either of which alone would be serious, had never till now been cured but by amputation.

The ligature of the principal artery of the limb, made at some distance from the wound, and between it and the heart, will I believe prevent the necessity of this cruel mutilation.'" 282.

Previous to making any comments on the preceding case and observations, Mr. Guthrie details the particulars of seven cases of wounded artery. We shall give a skeleton account of them. In the 1st the anterior tibial artery was wounded by a ball on the 16th of May. On the 15th of June secondary hemorrhage, for which the femoral artery was tied. Hemorrhage again took place on the 5th, 6th, and 27th of July—the limb was amputated, and the patient died. The muscles on the back of the leg were nearly gangrenous.

In the second case the wound was in the calf—secondary hemorrhage eight days afterwards with injection of the limb—femoral artery tied—hemorrhage again—amputation—death. The posterior tibial had been injured and sloughed.

In case 3, musket-ball passed through the thigh—aneurismal swelling—usual operation—matter collected in the thigh and a counter opening was made from which there was hemorrhage, which was arrested by pressure, returned, and amputation was performed. Patient died. The artery had not been wounded in the first instance, but become involved in the disease of the neighbouring parts.

In case 4, musket-ball entered a little in front of the left trochanter major, between the rectus and vastus externus, struck and flattened the os femoris, passed underneath the anterior edge of the glutæi and along the ilium for three inches, and lodged in the posterior part of the belly of the glutæus maximus, from whence it was cut out next day. Much blood was lost at the time—on the 15th day violent hemorrhage from the posterior wound, which on the employment of pressure, continued going on internally, and produced an aneurismal swelling—on the 18th day another profuse hemorrhage—incisions made to enlarge the wound, blood sponged out, two large branches of the gluteal artery tied at each extremity by the needle, and a large vessel close upon the bone which had furnished the bleedings treated in the same manner. The patient nearly sunk from exhaustion during the operation, and died the next day. No adhesion of the parts had taken place.

In case 5, a musket-ball broke both bones of the left leg—incisions were necessary—erysipelas and hospital gangrene followed—about a month after the injury hemorrhage from a spot two inches and a half above the ankle-joint. The anterior tibial was tied an inch and a half above the bleeding part, and all did well.

In case 6, a musket-ball wounded the left femoral artery a little below Poupart's ligament—on the 11th day slough separated from the wound with frightful hemorrhage. The external iliac was immediately tied with two ligatures and divided between them. Rigors, pain in the chest, and subsequently typhoid symptoms succeeded, but no return of bleeding; six days after the operation he died.

In case 7, a musket-ball entered the right leg in such a direction as to make it evident that it must have passed close to the posterior tibial and peroneal arteries, but no immediate hemorrhage ensued—thirteen days after the accident a considerable hemorrhage suppressed by the tourniquet, but shewing a constant disposition to recur. Next morning the limb was injected with blood, florid blood issued from both openings, and on passing the finger into the outer one a sort of aneurismal tumour could be felt, on pressing which against the fibula the hemorrhage ceased, indicating that the peroneal artery was in all probability the only vessel wounded. Mr. Guthrie cut down through the calf of the leg, found the parts in all states between sphacelus and perfect health, and after being obliged to make a transverse incision in addition to the longitudinal one of seven inches in length, succeeded in securing the vessel, but not separately, with a needle. The hemorrhage never returned, kindly suppuration took place, and in three months the wound was entirely healed. The patient is now in the York Hospital at Chelsea, and walks without appearing lame, although he cannot do so for any great distance. Having given the foregoing cases, which we have materially abridged, Mr. Guthrie proceeds to criticise the opinions of Baron Dupuytren, as appended to the case of M. de Gambaud.

"In the remarks which I am going to make on the memoir and opinions of M. Dupuytren, I hope that I shall not deviate from that respect which is due to a foreigner and a gentleman of high and deserved reputation. M. Dupuytren is pleased to entitle his operation after the method of Anel, although it was not done according to the method of Anel, but after that of Hunter; neither did Anel ever do an operation in the same place, or on the same principle. If the Baron had shown himself to be thoroughly acquainted with the principles that directed Mr. Hunter in the performance of his operation, I should have thought that the omission of his name might have arisen from a jealousy of his posthumous fame, unworthy of the elevated rank which M. Dupuytren himself holds in the profession; but it clearly arises from inattention to the principles laid down by Mr. Hunter and his successors, and to the difference which really exists between these operations.

It is also very extraordinary the Baron should say, as he does, page 22, that he had consulted in vain both ancient and modern authors on this subject, when the first and seventh of the cases I have given above were published, with several others nearly similar, in Paris, nine years before his memoir appeared, by Breschet in his translation of Mr. Hodgson's work on the Diseases of Arteries and Veins. It is nevertheless very satisfactory that it should be so, because it shows that the practice which M. Dupuytren recommmends to the French surgeons in 1828 as worthy of their adoption, had been tried in the British army in 1810 and 1812, and proved to be ineffectual, and to be founded on erroneous principles; whilst, in 1815, the true method of proceeding had been demonstrated by the same surgeons, and established on safe and scientific principles." 301.

This may be an unpalatable dose for the able Baron: but it is one which the state of his case most imperatively demands. He on all occasions evinces either an extraordinary ignorance, or an overweening contempt, of English surgery and English surgeons, a trait which, however it may flatter the reputed vanity of himself and his nation, lays him open to ridicule and bares his side to criticism. What can be more absurd than the omission of the name of John Hunter, in connexion with aneurism and operations on the arteries? What can be more foolish than the attempt to pluck the laurels that time has immoveably carved upon his bust, in order to deck the brows of Mr. Anel! The attempt is alike unsuccessful and pitiful, and only redounds to the disgrace and confusion of him who makes it. M. Dupuytren would appear to be like Horace, on his mistress' mention of his rival's rosy neck:—

——————Væ meum
Fervens difficili bile tumet jecur!

It gives us pain to allude in such terms to a surgeon of eminence and merit as M. Dupuytren confessedly is, but when our country is tacitly decried, and her great men robbed of their due, it becomes us to stand forward and maintain their rights. We would willingly believe that the spirit too frequently displayed by M. Dupuytren is not shared by the more liberal portion of his countrymen. But to revert from the man to his doctrines.

Mr. Guthrie proceeds to observe, that the last passage in M. Dupuytren's comments cannot be admitted as a correct statement of the effects of a gunshot wound on arteries.

"I have shewn in the preceding observations, pages 231 et seq. what is the real effect of a ball on the extremity of a divided artery, and that the appearances depend very much on the size and structure of the vessel. In what manner a ball can contract *(froncer)* the orifices of an artery has never been shewn, neither can it be easily understood; inasmuch as the act of contracting must be a vital act dependent on the powers of the artery itself. If it be a mechanical act, arising from injury, it must be a contusion; and this surely cannot be advanced as a process likely to consolidate the end of the vessel; it being now well known and admitted in England, that the first and most simple state of adhesive inflammation is the best calculated for the permanent closure of a divided artery." 203.

A torn, divided, or injured artery may suffer several different kinds of lesion; first, it may be only injured but not opened into; secondly, partly or entirely divided by a musket-ball: thirdly, it and the surrounding parts may be torn and contused by a cannon-shot. Thus, in reference to the first point the elasticity of arteries enables them to yield to an opposing force without laceration, and to suffer a considerable degree of contusion without sloughing. In the case of Captain Flack, several inches of the femoral artery were laid bare by a cannon shot, but it became covered by granulations, and maintained its functions unimpaired. If, however, the injury be considerable, a part or the whole of the circumference of the artery may ulcerate or slough.

If again an artery be completely divided, the appearances described by Mr. Guthrie in the commencement of this article will be observed. If only a part be torn, cut into, or slough, the patient will generally bleed to death, unless assistance be obtained; the musket or other ball having no effect on the walls of the artery different from any other instrument. Where a part of the vessel remains uncut, the opening made by the sword or ball, if small, becomes round from the unequal contraction of the artery. If the vessel be merely slit up the sides of the opening will come in contact, so as not to be very perceptible if the artery be compressed above, and no blood be allowed to pass through it. In such cases of imperfect division the only proper operation is to tie the vessel above and below.

When, in the third place, a cannon shot strikes a limb and bruises it most severely without carrying away any part, the great artery may not only be ruptured in one spot, but its internal coat may be injured in several. In one case related by Mr. Guthrie, the posterior tibial and fibular arteries were torn across, and the popliteal was closed by coagulable lymph thrown out from a rupture of the internal coat at this part. If an artery be wounded in man by a sharp cutting instrument, to the extent of one-fourth of its circumference, or even less, Mr. Guthrie believes that the process of cure always takes place through inflammation, and by obliteration of that part of the canal of the vessel. In proof of this he mentions a case of pike-wound in the direction of the brachial artery, after which no pulse could be felt for some time at the wrist, and the pulsation of the brachial below the wound continues imperfect.

"I consider myself then warranted in saying, first, that when an artery is injured by a ball, but not torn or bruised to such an extent as to destroy the continuity of the vessel, inflammation is the only result; secondly, that when the artery is cut or divided, the processes I have described take place; thirdly, that in some cases, particularly where the injury is inflicted by cannon shot, the internal coat may be torn in one or more places above the part where it is divided, constituting a barrier to the flow of blood from the part; but it must be recollected that this barrier is formed at a very early period. I much doubt whether blood would pass through such an artery: I am sure that it would not do so twenty-four hours after the injury. The impulse or power of the heart or circulation is as nothing when the inner coat of an artery is injured, and has inflamed so as to throw out coagulable lymph. It invariably arrests the circulation and obliterates the artery.

In all cases in which an artery is seen pulsating on the surface or other part of a wound, the obstacle to the bleeding is found to exist in the very extremity of the vessel: let only one-sixteenth part of an inch be cut off, and the blood immediately darts forth. I have done this fifty times at least, and it is precisely the same from whatever cause the injury is inflicted.

A divided or cut artery, in a case of wound by a musket ball, is not in a more favourable state for healing without hemorrhage than from any other wound. In regard to the other observation of M. Dupuytren, that a gun-shot wound has the remarkable property of concreting and coagulating the blood in their extremities, in a greater degree than any other wound, it is contrary to every fact I am acquainted with in regard to large arteries. The theory then which he would build on these opinions is untenable. If the wound had such an effect on the artery, why did it bleed at first? why did it continue to do so afterwards? I have shown that the lower end of the artery is more likely to bleed than the upper, and that hemorrhage does not always depend on the impulse of the circulation. It is certainly true that if the blood can be prevented from passing into the divided vessel, there will be a greater chance of the natural processes of inflammation and granulation, which are taking place in and around it, closing it up, than if the blood be allowed to flow through it. But it is only then a chance. It is impossible to calculate the time which nature may require to bring the blood by the collateral vessels into either the upper or lower end of the vessel; it may occur immediately; it may not do so for hours or for days; and on the speculation that it may not do so, the first hope of safety depends; the second, on the further accidental circumstance, that the end of the artery may be closed in the interval. Surely this cannot be considered a scientific operation, and fit to be erected into a precept in surgery, which depends on two accidental circumstances, neither of which can in the slightest degree be calculated upon.

There are many other reasons why the operation was a bad one in this case, and always will be a bad one in all similar cases. The patient was made to undergo the chance of mortification of the extremity, which it is probable would have taken place, if the operation had not been delayed until some days after the first hæmorrhage occurred from the wound; which did not take place until the thirteenth day, during which time the inflammation in the limb had given the collateral vessels a disposition to enlarge. The wound itself was not treated as the principles of surgery require. A quantity of decomposed blood was pent up under and between the muscles of the calf of the leg, together with some of the patient's clothes, and some spiculæ of bone. Surely in a case like this, but without further fear of hemorrhage, the Baron would have enlarged the wound, cleared away the clots of blood, and have placed it in a simple state. There cannot be a doubt on the subject, and the operation of making an incision through the muscles of the calf of the leg would have enabled him to do all this, and to have secured the vessels, if there had been even four bleeding extremities, without any difficulty or danger.

The Baron Dupuytren applied Mr. Hunter's theory of the operation for aneurism to the treatment of a wounded artery, and succeeded by chance; others have done the same long before him; but nothing which is dependent on chance or accident can ever become a principle in surgery." 310.

The theory, observes Mr. G. is not always applicable even when the wounded artery forms an aneurism, because the whole limb is not in the same state as one which has gradually become aneurismal from disease. A man, aged 24, was wounded in the thigh on the 18th of June, and lost much blood. The wound healed, but an aneurism formed and extended to within an inch of Poupart's ligament. On the 28th of August staff-surgeon Collier tied the external iliac artery, in the manner recommended by Sir Astley Cooper. The temperature of the limb shewed a great disposition so sink, discoloured patches appeared upon its surface, and pain in the abdomen with inflammation of the wound succeeded. The patient was bled at three times to 36 ounces, the limb turned livid and vesicated, and on the 1st of September the patient died. On dissection there was slight peritoneal inflammation, and the whole limb was in a state of gangrene. The operator, says Mr. Guthrie, should either have cut into the sac and performed the old operation, or he should have tied the artery immediately above it. More anastomotic branches would thus have been saved, and mortification probably averted.

When a deep seated branch of an artery is wounded and continues to bleed, considerable difficulty often arises as to the best method of proceeding; because it is possibly an uncertain branch, and the facility of anastomosis must be taken into account. It is not good practice to cut down upon an artery on the first hæmorrhage, unless the main trunk be wounded; for many a bleeding supposed to come from the great vessel, has been permanently stopped by a moderately continued pressure in the course of the vessel, sometimes combined with pressure on the bleeding part itself. If, however, this be insufficient, Mr. G. would introduce his finger into the wound, and enlage it until he could see to the bottom or the bleeding part, when he would tie the two extremities of the vessel.

Hæmorrhage may take place from an artery which cannot be tied at the part where it is wounded, as in the throat; and the question of placing a ligature on the main trunk comes under consideration. Mr. Guthrie details a case, in which both carotid arteries were wounded by pins, purposely introduced on a cork, which stuck in, and produced ulceration of the œsophagus. He also relates a case which occurred to staff-surgeon Collier, in which a spear wound at the angle of the jaw, was followed by secondary hæmorrhage, and the common carotid artery on the same side was tied with success. He then adverts to the cases of Mr. Luke and Mr. Mayo, in which the same operation was successfully performed, for hæmorrhage in consequence of ulceration of the throat.* Our author was summoned to see a gentleman who had cut his throat very deeply, having laid bare the left carotid artery, and wounded the left internal jugular vein. The opening in the latter was distinct, and Mr. Guthrie ripped up the edges and included them in a knot, without destroying the continuity of the vessel. The carotid appeared to be wounded as deeply as its inner coat, but no deeper. On the eighth day secondary hæmorrhage took place from the artery, and Mr. Guthrie tied it below, and the external carotid above the wound; the bleeding ceased, but the patient died next day. The internal jugular was found pervious, and the internal carotid had a soft coagulum of blood for about a quarter of an inch. In a similar case Mr. Guthrie, warned by this, would place a ligature above and below the injury to the outer coat of the vessel.

In all cases of hæmorrhage from the throat, which cannot be suppressed without tying the carotid artery, Mr G. would tie the external, as being nearer the bleeding branch. If this fails, he would tie the internal or common carotid also.

In bleeding wounds of the hand or foot, dilate the wound and tie the vessel, or if this is impracticable or unsuccessful, compress the principal trunks and the wound itself. The ulnar artery in the palm should always be tied when wounded, for the deeper seated radial, compression should be tried. If this be not allowed by the swelling of the hand, first tie the radial, then the ulnar, and if these operations fail, a clean and decided incision is to be made in the line of the wound, from the annular ligament to the finger (avoiding the flexor tendons), and down to the metacarpal bone, which bone, and the finger is, *if necessary*, to be removed; by which space will be obtained to see the bleeding vessels. The hand or foot should only be amputated as the very last resource. Such are the rules laid down by Mr. Guthrie in cases of wounds of the hand or foot. Some very good remarks are offered on wound of the brachial artery in bleeding, and then the precepts already inculcated in different parts of this article are summed up together, in the form of 24 aphorisms or "conclusions." We wish we had space for their insertion. A chapter on aneurism by anastomosis, and one on the mode of performing the various operations on the arteries, conclude this valuable work.

Here we must end, not for want of matter, but deficiency of room. After the copious account we have given of a portion of Mr. Guthrie's book, it would be a work of supererogation to say that we think it deserving of very attentive perusal. Surgeons will derive much information and correct principles from studying its contents, which we recommend all to do. In our next Number we shall analyze that portion which treats of the diseases of arteries.

---

* We have given a full account of these and other cases in the Periscope of the present Number, p. 260, et seq.

